# DNA Methylation Analysis of Bone Marrow Cells at Diagnosis of Acute Lymphoblastic Leukemia and at Remission

**DOI:** 10.1371/journal.pone.0034513

**Published:** 2012-04-06

**Authors:** Jessica Nordlund, Lili Milani, Anders Lundmark, Gudmar Lönnerholm, Ann-Christine Syvänen

**Affiliations:** 1 Department of Medical Sciences, Molecular Medicine, Uppsala University, Uppsala, Sweden; 2 Department of Women's and Children's Health, Uppsala University, Uppsala, Sweden; The University of Arizona, United States of America

## Abstract

To detect genes with CpG sites that display methylation patterns that are characteristic of acute lymphoblastic leukemia (ALL) cells, we compared the methylation patterns of cells taken at diagnosis from 20 patients with pediatric ALL to the methylation patterns in mononuclear cells from bone marrow of the same patients during remission and in non-leukemic control cells from bone marrow or blood. Using a custom-designed assay, we measured the methylation levels of 1,320 CpG sites in regulatory regions of 413 genes that were analyzed because they display allele-specific gene expression (ASE) in ALL cells. The rationale for our selection of CpG sites was that ASE could be the result of allele-specific methylation in the promoter regions of the genes. We found that the ALL cells had methylation profiles that allowed distinction between ALL cells and control cells. Using stringent criteria for calling differential methylation, we identified 28 CpG sites in 24 genes with recurrent differences in their methylation levels between ALL cells and control cells. Twenty of the differentially methylated genes were hypermethylated in the ALL cells, and as many as nine of them (*AMICA1, CPNE7, CR1, DBC1, EYA4, LGALS8, RYR3, UQCRFS1, WDR35*) have functions in cell signaling and/or apoptosis. The methylation levels of a subset of the genes were consistent with an inverse relationship with the mRNA expression levels in a large number of ALL cells from published data sets, supporting a potential biological effect of the methylation signatures and their application for diagnostic purposes.

## Introduction

Acute lymphoblastic leukemia (ALL) is the most common childhood malignancy accounting for 25% of all childhood cancers in developed countries. ALL originates from the malignant transformation of lymphocyte progenitor cells into leukemic cells in the B-cell and T-cell lineages [1]. However, most of the known large scale genetic aberrations in ALL are not alone sufficient to induce the disease [2], suggesting that there are other genetic or epigenetic alterations that act in leukemic transformation.

In mammalian genomes, methylation of the C-residue in CpG dinucleotides plays an important role in regulating gene expression [3], [4]. DNA methylation is maintained by DNA methyltransferases (DNMTs). Alterations in the expression of DNMTs in blood progenitor cells results in extensive changes in methylation patterns, which may lead to leukemogenesis [5]. Treatment with inhibitors of DNA methylation, such as 5′-azacytidine have therapeutic benefits in leukemia [6], indicating that the methylation changes are functionally important. In cancer, the regions near transcription start sites often show increased methylation levels, as opposed to an overall decrease in DNA methylation on the genome-wide level [7], [8], [9]. DNA hypermethylation in the promoters of putative tumor suppressor genes has been found to correlate with resistance against chemotherapy in ALL [10]. We and others have shown that the methylation levels of sets of genes have potential as prognostic markers for risk of relapse in pediatric ALL [11], [12]. Moreover, two studies have suggested that minimal residual disease in leukemia patients can be detected by the methylation status of only a few genes [13], [14]. Thus, epigenetic perturbation of DNA methylation can be a valuable source of information for understanding the biology of gene regulation, phenotypic diversity, and treatment outcome in pediatric ALL.

In a previous genome-wide survey of 8,000 genes in 197 bone marrow or blood samples from patients with pediatric ALL, we identified >400 genes that displayed allele-specific gene expression (ASE) [4]. The observed ASE indicates that the expression of these genes could be regulated by DNA methylation that silences or activates gene expression in an allele-specific manner. The methylation pattern of the genes with ASE allowed classification of ALL subtypes and stratification of patients into prognostic subgroups [11]. In the current study, we hypothesized that the selection of genes based on genome-wide ASE analysis would enrich for genes with functional CpG site methylation that could be involved in the pathogenesis of ALL. Our aim was to identify genes that display aberrant DNA methylation independently of cytogenetic ALL subtype for further mechanistic studies of ALL. We investigated how the methylation status of the 1,320 CpG sites in genes with ASE differs between ALL samples taken at diagnosis and matched bone marrow samples from the same patients during and after induction therapy, when the patients were in remission, and in control cells from bone marrow or blood of non-leukemic individuals.

## Materials and Methods

### Samples from patients and controls

Mononuclear cells were isolated from bone marrow aspirates or peripheral blood cells by 1.077 g/mL Ficoll-Isopaque (Pharmacia) density-gradient centrifugation from 63 samples. The samples consisted of 20 bone marrow samples taken at diagnosis of ALL, 30 follow-up samples from bone marrow samples taken from the same patients during therapy, and 13 non-leukemic control samples, of which 11 were from bone marrow and two were from peripheral blood of children the same age as the patients. The clinical and cytogenetic information for the patients is provided in **Table 1**. The patients were treated according to the ALL 2000 protocol of the Nordic Society of Pediatric Oncology (NOPHO) [15], in which no DNA-demethylating drugs are used. The proportion of leukemic cells was estimated in each sample by light microscopy in May-Grünwald-Giemsa–stained cytocentrifugate preparations. The proportion of leukemic blasts exceeded 90% in the ALL samples included in this study. The matched patient samples taken during therapy at days 29, 50 and 106 contained less than 5% leukemic blasts, indicating that the patients were in morphological remission. The non-leukemic control cells were obtained from sex- and age-matched pediatric patients with an initial suspicion of leukemia, from which an initial ALL diagnosis was excluded by negative diagnostic tests and clinical follow-up (**Table 1**). DNA was extracted from cell pellets by the AllPrep DNA/RNA Mini Kit (Qiagen) or the QIAamp DNA Blood Mini Kit (Qiagen). The Regional Ethics Committee in Uppsala, Sweden approved the study, and the patients and/or their guardians provided written informed consent. The study was conducted in accordance with the Declaration of Helsinki.

**Table 1:** Clinical information for the 20 patients with acute lymphoblastic leukemia and 13 controls included in the study.

Patient ID	Immuno-phenotype	Genetic subtype	Age at diagnosis, years	sex	WBC count^a^	NOPHO treatment protocol^b^	Remission samples^c^
Patient_1	BCP	t(4;11)(q21;q23)	0.8	female	99.7	infant	50
Patient_2	BCP	amp(21)	4.3	male	11.6	IR	106
Patient_3	BCP	amp(21)	5.9	female	4.3	SR	106
Patient_4	BCP	HeH	3.3	male	95.0	HR	106
Patient_5	BCP	HeH	6	male	11.2	IR	29, 50,106
Patient_6	BCP	HeH	2.6	male	7.2	SR	29, 50,106
Patient_7	BCP	HeH	3.5	male	5.0	HR	106
Patient_8	BCP	HeH	3.8	male	3.0	SR	29, 50,106
Patient_9	BCP	HeH	14.1	female	24.5	IR	29, 50,106
Patient_10	BCP	HeH	1.9	male	39.6	HR	29, 50,106
Patient_11	BCP	unknown	5.5	female	15.2	HR	106
Patient_12	BCP	unknown	12	male	43.9	HR	106
Patient_13	BCP	normal	13.2	male	24.0	IR	106
Patient_14	BCP	t(12;21) (p13;q22)	6.2	female	4.2	SR	106
Patient_15	BCP	t(12;21) (p13;q22)	3.7	male	12.3	IR	50
Patient_16	BCP	t(9;22) (q34;q11)	11.2	male	64.4	HR	106
Patient_17	T-ALL	T-ALL	13.9	female	139.0	HR	106
Patient_18	T-ALL	T-ALL	10.3	male	244.0	HR	106
Patient_19	T-ALL	T-ALL	4.3	male	107.0	HR	50
Patient_20	T-ALL	T-ALL	7.7	male	44.4	HR	50
Non-Leukemic 1	NA	NA	4.1	female	NA	NA	NA
Non-Leukemic 2	NA	NA	0.2	male	NA	NA	NA
Non-Leukemic 3	NA	NA	0.6	female	NA	NA	NA
Non-Leukemic 4	NA	NA	8.3	female	NA	NA	NA
Non-Leukemic 5	NA	NA	6.9	female	NA	NA	NA
Non-Leukemic 6	NA	NA	14.7	male	NA	NA	NA
Non-Leukemic 7	NA	NA	0.9	male	NA	NA	NA
Non-Leukemic 8	NA	NA	15.3	male	NA	NA	NA
Non-Leukemic 9	NA	NA	14.4	male	NA	NA	NA
Non-Leukemic 10	NA	NA	5.1	male	NA	NA	NA
Non-Leukemic 11	NA	NA	4.0	female	NA	NA	NA
Non-Leukemic 12	NA	NA	14.3	female	NA	NA	NA
Non-Leukemic 13	NA	NA	1.1	female	NA	NA	NA

BCP indicates B-cell precursor ALL; T-ALL, T-cell ALL; HeH, high hyperdiploidy; amp(21), amplification of chr 21; HR, high risk; SR, standard risk; IR, intermediate risk; NA, not available.

a White blood cell count at diagnosis (10^9^/L).

b The NOPHO ALL 2000 protocol was used.

c DNA from was available from bone marrow taken from the patients on day 29,50, and/or 106 after the initiation of therapy, all patients were in morphological remission with less than 5% leukemic blasts.

### DNA methylation analysis

A custom-designed panel of CpG sites was analyzed to determine the methylation levels of 1,536 CpG sites located 2 kb upstream to 1 kb downstream of the transcription start site of 416 genes [4]. Six hundred ng of genomic DNA was treated with sodium bisulfite (EZ-96 DNA Methylation Kit, Zymo Research) for subsequent genotyping by the Golden Gate Assay (Illumina Inc.). The methylation level of each CpG site is obtained from the genotyping assay as a β-value ranging from 0.0–1.0, which corresponds to no methylation of either allele to complete methylation of both alleles of the analyzed genes. Genotyping and quality control were performed as previously described [11]. After quality filtering, there were 1,320 CpG sites distributed over 413 gene regions, with 1–10 CpG sites per gene, remaining for analysis (Table S1). We previously reported that the concordance between the methylation levels determined by the Golden Gate assay and by Sanger sequencing of bisulfite-converted DNA for five randomly selected CpG sites was 87% [11] (Figure S1). Moreover, the concordance between the methylation levels of 21 ALL samples run in replicate using the GoldenGate Assay was high, with a median site-wise Pearson correlation coefficient R = 0.88 for the 28 CpG sites highlighted in the present study (Table S2).

### Gene expression data

Genome-wide gene expression data was retrieved from two ALL datasets via the Oncomine tool (Compendia Bioscience). The first dataset contained expression data for 98 ALL patients and bone marrow cells from six healthy controls [16]. The second dataset contained expression data for 533 ALL patients and PBMCs from 74 healthy controls [17].

### Statistical analyses

The similarity of individual methylation profiles was assessed using the Pearson correlation coefficient (*R*). Hierarchical clustering was performed by “hclust” with one minus the correlation coefficient as the similarity measure for individual samples and between individual CpG sites. The Wilcoxon Signed-Rank test was used to identify CpG sites with differences in methylation between the paired diagnostic and remission samples. The Wilcoxon Rank-Sum test was used to test for differentially methylated CpG sites between diagnostic BCP and T-ALL samples. Where indicated, *P*-values were adjusted for multiple testing with the Benjamini-Hochberg method. The Friedman's test was used to identify CpG sites with differential methylation in serial bone marrow samples taken from the same individuals. All statistical analyses were performed in R. Gene lists were analyzed by Ingenuity Pathway Analysis (IPA) (Ingenuity® Systems). Pathway-associated *P*-values were calculated with a Fisher's exact test. The *P*-value is based on the enrichment of differentially methylated genes compared to the 413 genes with ASE that were analyzed.

## Results

### Analysis of differential DNA methylation between diagnostic ALL samples, remission samples, and controls

**Figure 1:**
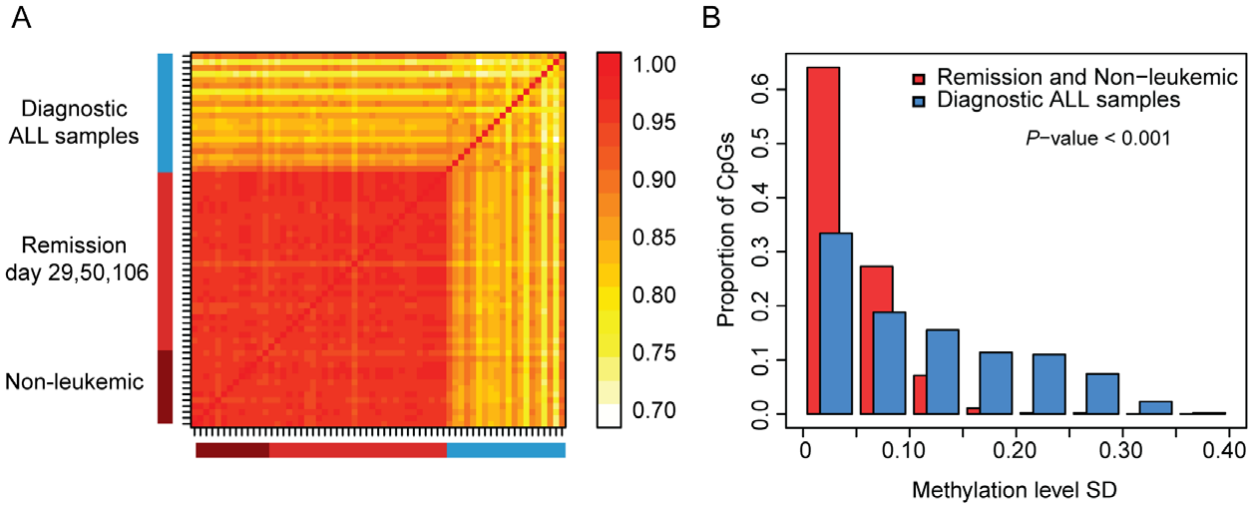
Correlation matrix and variability of the methylation levels measured at 1,320 CpG sites across the 63 samples included in the study. (A) Each individual sample is indicated by a black line on the axes. The methylation levels in the samples taken at remission during induction therapy at day 29 and during consolidation therapy at days 50 and 106 are highly correlated with the methylation levels in the non-leukemic samples (median Pearson's correlation coefficient (*R*) = 0.96), while the diagnostic ALL samples are less similar both to each other and to the samples taken after treatment, and to the non-leukemic samples (median *R* = 0.83). The scale for the correlation coefficients is shown to the right of the matrix. The red color indicates higher correlation (greater similarity), while the light yellow indicates less correlation (less similarity). (B) Histograms of the standard deviations (SD) for the methylation levels measured for 1,320 CpG sites across 20 ALL samples (blue) and across the combined 33 remission samples and 13 non-leukemic controls (red). SD bins are shown on the horizontal axis. The vertical bars show the proportion of observations in each SD bin. The CpG sites show greater variability in the ALL samples than in the remission samples and non-leukemic controls (Wilcoxon Rank-Sum P<0.001).

To identify genes with differential DNA methylation, we compared the methylation levels of 1,320 CpG sites in mononuclear cells from bone marrow taken at the time of ALL diagnosis to bone marrow mononuclear cells from the same patients at day 29, 50 or 106 of therapy, when the patients were in remission, and to bone marrow and peripheral blood mononuclear cells from non-leukemic controls. The data for the 1,320 CpG sites from all samples is available in the Supporting Information (Table S1). We found that the methylation pattern across the 1,320 CpG sites in each of the bone marrow samples of ALL patients were distinct from the samples taken at remission and from the non-leukemic controls (**Figure 1A**). The methylation levels of each individual CpG site displayed low variability between samples with a mean standard deviation (SD) of 0.045 across all the 1,320 CpG sites in the DNA samples taken at remission and in the DNA samples from the non-leukemic controls. In contrast, the methylation levels of the CpG site displayed higher variability between samples across the 1,320 CpG sites (mean SD = 0.12) in the ALL cells taken at diagnosis (**Figure 1B**). We did not detect any statistically significant differences (Permuted Friedman's *P*<0.01 and Δβ>0.10) when the methylation levels of the DNA samples from five ALL patients collected at different time points during remission were compared group-wise (day 29, 50, 106). The small sample size in this analysis precludes detection of statistically significant differences, but we cannot exclude the possibility that there might be differences in CpG site methylation levels between samples at different time points during induction treatment.

**Figure 2:**
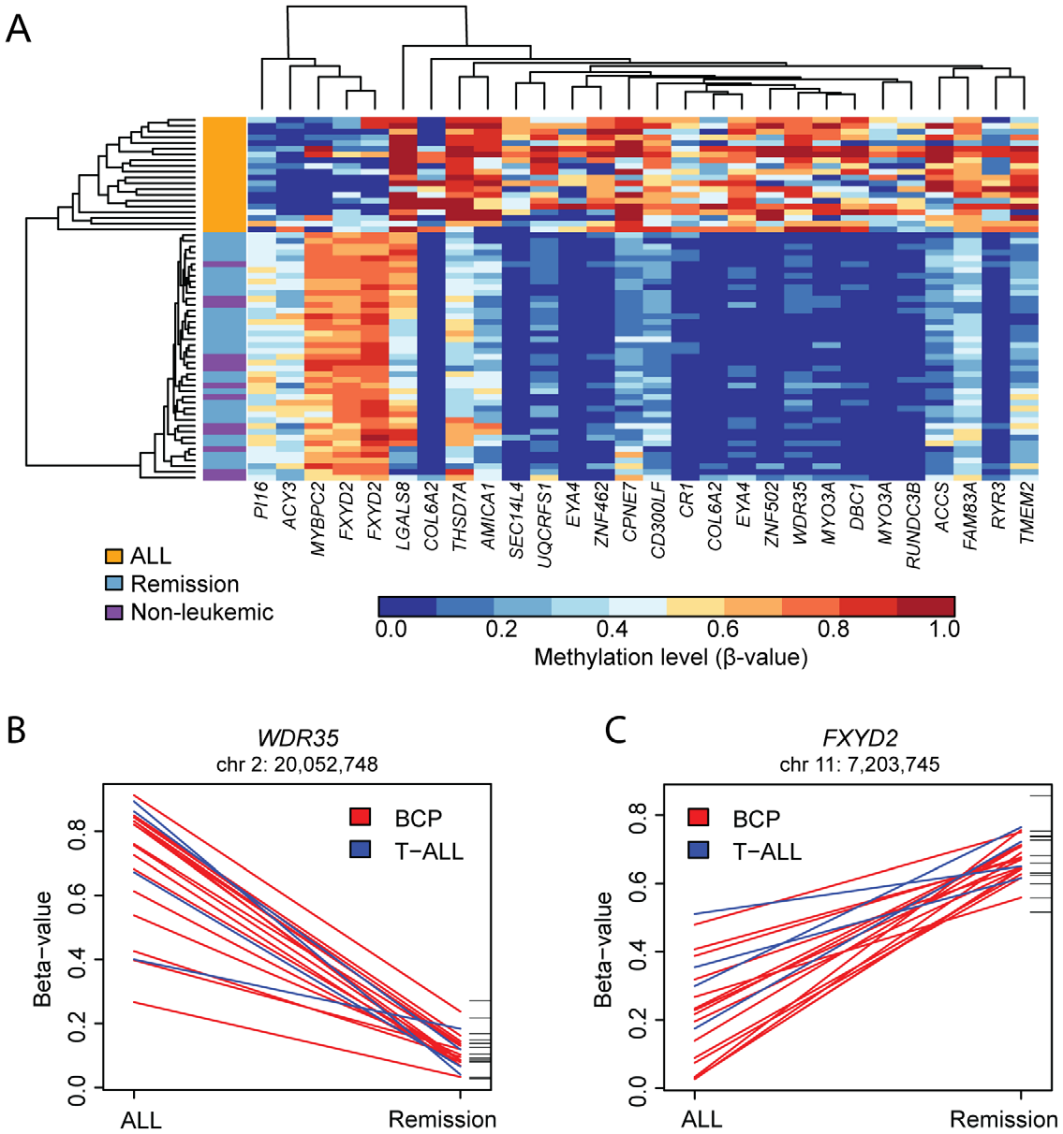
Differential methylation in ALL cells. (A) Heatmap of the methylation profiles of the 28 CpG sites that are differentially methylated between the diagnostic ALL samples, bone marrow cells at remission and non-leukemic bone marrow cells. The ALL samples (orange) and bone marrow cells during remission (blue) form two distinct groups. Thirteen bone marrow cell samples from non-leukemic controls (purple) cluster among the samples collected during remission. The scale for the methylation β-values is shown below the heatmap. The elongated heights of the dendrogram branches between the ALL samples compared to the normal samples illustrate the increased variability in the ALL samples for the 28 CpG sites. Graphs showing the differences in methylation level between CpG sites in the (B) *WDR35* and (C) *FXYD2* genes at the time of diagnosis (left vertical axis) and during remission (right vertical axis). The data points for each paired sample are connected with a red line for B-cell precursor (BCP) samples and with a blue line for T-ALL samples. The corresponding CpG methylation levels in 13 non-leukemic control samples are shown as black horizontal lines to the right of the graphs. The CpG site at chr2:20,052,748 in the *WDR35* gene (B) was hypermethylated in diagnostic ALL samples and hypomethylated at remission and in non-leukemic controls, while the CpG site at chr11:7,203,745 in the *FXYD2* gene (C) displayed the opposite pattern. The BCP and T-ALL samples display the same pattern of methylation difference in these two genes.

**Figure 3:**
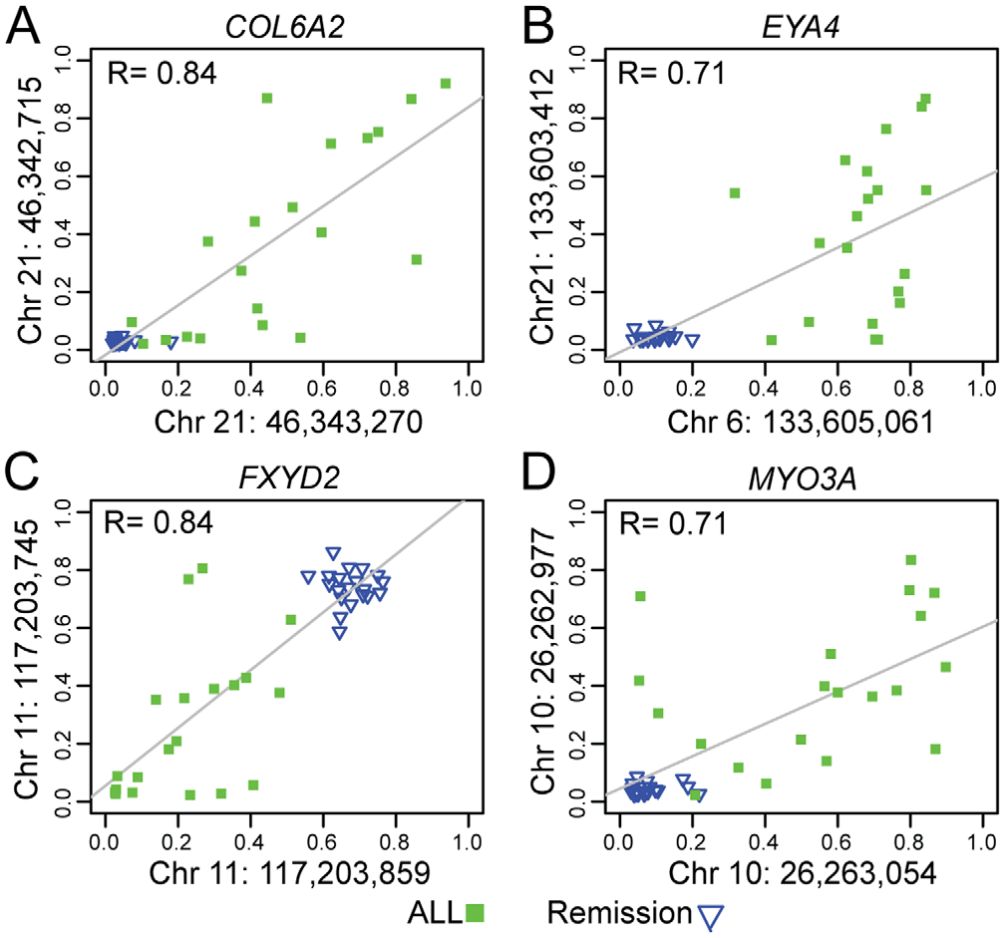
Correlation between the methylation levels (β-values) of two CpG sites located in the *COL6A2*, *EYA4*, *FXYD2* and *MYO3A* genes. The Pearson's correlation coefficients (*R*) across the 20 acute lymphoblastic leukemia (ALL) samples taken at ALL diagnosis (green) and the 20 matched bone marrow samples taken at remission (blue) for the four genes are shown in panels A–D. The positions of the CpG sites for which the β-values are plotted are indicated on the axes in each panel (Human Genome Build 36). The inter-individual variation between the pairs of CpG sites in the remission cells is consistently lower than between the ALL cells, which speaks against the variation in ALL cells arising because of methodological factors.

We applied stringent criteria for detecting CpG sites with differential methylation between the cells at ALL diagnosis and bone marrow cells at remission, by requiring a adjusted *P*-value<0.001 for the median difference in Δβ-values between the two groups and a threshold of 0.30 for calling a CpG site as differentially methylated. This analysis identified 28 CpG sites in 24 genes with differential methylation between the cells taken at ALL diagnosis and bone marrow mononuclear cells at remission (**Table 2**). A large proportion (45–95%) of the individual sample pairs fulfilled the criterion of a Δβ-value>0.3 for the 28 CpG sites. Hierarchical clustering of the samples at diagnosis (n = 20), at remission (n = 30) and the non-leukemic control cells (n = 13) according to the methylation levels of the 28 differentially methylated CpG sites resulted in unequivocal separation between the ALL samples and the bone marrow samples at remission (**Figure 2A**), with the non-leukemic control samples clustering together with the samples taken at remission. The CpG sites displayed two distinct patterns of differential methylation. For 23 of the 28 CpG sites, exemplified by a CpG site in the *WDR35* gene (**Figure 2B**), the methylation levels were higher in the ALL cells at diagnosis than in the bone marrow cells during remission (median Δβ = 0.66). We also identified five CpG sites with the opposite pattern, like *FXYD2* (**Figure 2C**), with higher median methylation levels in the cells at remission (median Δβ = 0.55). Four of the genes with differential methylation according to the stringent criteria applied (*COL6A2*, *EYA4*, *FXYD2*, *MYO3A*) contained two differentially methylated CpG sites. The methylation levels (β-values) of the CpG sites in these genes were correlated (*R*>0.70) (**Figure 3**). At less stringent criteria for calling differential methylation (*P*<0.05 and Δβ>0.2) the methylation status of 1–2 additional CpG sites in nine of the genes supported the corresponding hyper- or hypomethyation (Table S1).

**Table 2:** CpG sites with differential methylation between acute lymphoblastic leukemia cells and remission cells.

				Median β-value (range)					
Gene symbol^a^	CpG site location^b^		Distance from TSS^c^	ALL diagnosis	Remission	Median Δβ-value^d^	N Δβ-value>0.3^e^	Adjusted P-value^f^	mRNA expression^g^
*ACCS*	11p11	44,044,910	477	0.58 (0.03–0.92)	0.18 (0.09–0.49)	0.41	12	4.30E-04	nc
*ACY3*	11q13	67,174,534	172	0.04 (0.02–0.69)	0.41 (0.18–0.67)	−0.37	12	1.94E-04	+[16]
*AMICA1*	11q23.3	117,602,131	1,204	0.80 (0.03–0.94)	0.35 (0.07–0.57)	0.45	15	6.53E-04	−[16] [17]
*CD300LF*	17q25.2	70,220,061	642	0.67 (0.35–0.85)	0.18 (0.04–0.44)	0.49	15	1.48E-04	−[16] [17]
*COL6A2*	21q22.3	46,343,270	800	0.44 (0.07–0.94)	0.03 (0.02–0.18)	0.41	14	1.48E-04	nc
*COL6A2*	21q22.3	46,342,715	245	0.34 (0.02–0.92)	0.03 (0.02–0.05)	0.32	10	1.94E-04	nc
*CPNE7*	16q24.3	88,170,539	862	0.86 (0.28–0.95)	0.25 (0.12–0.67)	0.61	15	1.94E-04	nc
*CR1*	1q32	205,736,601	476	0.46 (0.19–0.75)	0.04 (0.02–0.24)	0.41	13	1.48E-04	−[17]
*DBC1* *	9q32-q33	121,170,638	884	0.61 (0.06–0.93)	0.05 (0.03–0.13)	0.56	15	1.48E-04	−[16]
*EYA4*	6q23	133,605,061	855	0.70 (0.32–0.84)	0.10 (0.04–0.20)	0.60	19	1.48E-04	nc
*EYA4*	6q23	133,603,412	−794	0.42 (0.03–0.87)	0.04 (0.04–0.09)	0.37	12	6.53E-04	nc
*FAM83A*	8q24.13	124,263,705	−228	0.66 (0.25–0.82)	0.36 (0.17–0.57)	0.31	11	5.36E-04	nc
*FXYD2*	11q23	117,203,859	158	0.22 (0.03–0.51)	0.67 (0.56–0.76)	−0.45	14	1.48E-04	+[16] [17]
*FXYD2*	11q23	117,203,745	272	0.20 (0.02–0.81)	0.75 (0.59–0.86)	−0.55	16	3.93E-04	+[16] [17]
*LGALS8*	1q43	234,751,397	−1,963	0.89 (0.35–0.94)	0.49 (0.19–0.85)	0.39	10	5.36E-04	nc
*MYBPC2*	19q13.33	55,628,143	139	0.06 (0.02–0.88)	0.67 (0.57–0.78)	−0.61	15	6.53E-04	+[16]
*MYO3A*	10p11.1	26,263,054	148	0.57 (0.05–0.90)	0.07 (0.03–0.22)	0.51	14	1.48E-04	nc
*MYO3A*	10p11.1	26,262,977	−225	0.38 (0.02–0.83)	0.04 (0.03–0.09)	0.34	12	2.70E-04	nc
*PI16*	6p21.31	37,029,477	−710	0.14 (0.03–0.64)	0.44 (0.29–0.59)	−0.31	9	4.30E-04	nc
*RUNDC3B* *	7q21.12	87,096,478	813	0.45 (0.04–0.80)	0.07 (0.03–0.15)	0.38	13	4.30E-04	nc
*RYR3*	15q14-q15	31,390,843	374	0.39 (0.06–0.89)	0.07 (0.03–0.18)	0.33	10	1.48E-04	nc
*SEC14L4*	22q12.1	29,231,446	236	0.44 (0.02–0.72)	0.05 (0.02–0.23)	0.40	12	8.11E-04	−[17]
*THSD7A*	7p21.3	11,840,245	−1,902	0.83 (0.42–0.93)	0.45 (0.19–0.69)	0.39	12	1.48E-04	nc
*TMEM2*	9q13-q21	73,572,286	942	0.77 (0.04–0.89)	0.22 (0.06–0.49)	0.54	13	4.30E-04	−[16]
*UQCRFS1*	19q12	34,395,007	−947	0.66 (0.02–0.95)	0.14 (0.02–0.35)	0.51	15	4.30E-04	nc
*WDR35*	2p24.3	20,052,748	−617	0.76 (0.27–0.91)	0.10 (0.03–0.24)	0.66	17	1.48E-04	nc
*ZNF462*	9q31.3	108,663,645	−1,554	0.66 (0.05–0.90)	0.06 (0.05–0.26)	0.60	16	4.30E-04	nc
*ZNF502*	3p21.32	44,729,363	221	0.62 (0.11–0.93)	0.03 (0.02–0.22)	0.59	15	1.48E-04	nc

a Gene symbol according to the HUGO Gene Nomenclature Committee (http://www.genenames.org/);

* indicates genes selected from the literature;DBC1 [24]; RUNDC3B [25].

b Chromosome number and coordinate of the CpG site (Human genome build 36).

c Distance from the transcription start site (TSS); −, upstream from the TSS; +, downstream from the TSS.

d Median difference in beta-value between ALL patients at diagnosis and remission for paired samples (ALL-remission).

e Number of ALL-remission pairs with Δβ-values larger than 0.30.

f Adjusted Wilcoxon Signed-Rank *P*-values corrected for multiple testing with the Benjamini Hochberg approach.

g Genes up (+) or down (−) regulated in ALL cells compared to controls according to published datasets [16], [17], n.c. = no change.

The CpG site in the *MYBPC2* gene was differentially methylated (Wilcoxon Rank-Sum test, *P*-value<0.001) between ALL cells of B-cell origin (BCP ALL, n = 16) and T-cell origin (T-ALL, n = 4), with hypomethylation in BCP ALL (median β-value = 0.04) and hypermethylation in T-ALL (median β-value = 0.75). The other 27 CpG sites did not display differential methylation between BCP and T-ALL samples (Table S2), indicating that the majority of the genes identified here based on their methylation profiles are characteristic for ALL cells, independently of immuno-phenotype.

### Regulation of gene expression by DNA methylation

On a genome-wide scale there is an inverse relationship between DNA methylation in the vicinity of the TSS and mRNA expression [18]. To examine whether the differentially methylated CpG sites identified here had potential regulatory functions, we queried two published sets of mRNA expression data from ALL cells with data for 98 and 533 ALL samples, respectively [16], [17], for up- or down-regulation of the differentially methylated genes. In these datasets, the *AMICA1*, *DBC1*, *CD300LF*, *CR1*, *SEC14L4* and *TMEM2* genes identified in our study as hypermethylated were down-regulated and the hypomethylated genes *ACY3*, *FXYD2*, and *MYBPC2* were up-regulated with 2-fold differences in expression levels between ALL cells and control bone marrow cells [16] or peripheral blood mononuclear cells from healthy individuals [17] (**Table 2**) in at least one dataset. The other genes identified in our differential methylation analysis did not meet the minimum criteria of 2-fold differential expression.

### Biological roles for the genes with differential methylation

The 24 differentially methylated genes highlighted in our study (**Table 2**) were enriched (*P*<0.05) for functions such as cell-to-cell signaling and interaction (*AMICA1, CR1, LGALS8, RYR3*) and cell death/apoptosis (*CR1, DBC1, EYA4, LGALS8, UQCRFS1*) (**Table 3**). Among the differentially methylated genes, several have been previously identified as differentially methylated in cancer and are known to be involved in ALL. *EYA4* is frequently hypermethylated and down-regulated in colon and esophageal cancers [19], [20]. Expression of *LGALS8* and *UQCRFS1* are associated with relapse in T-ALL [21], [22] and the *COL6A2*, *DBC1* and *RUNDC3B* genes have been found to be hypermethylated and down-regulated in pediatric ALL samples [23], [24], [25]. The *AMICA1* and *FXYD2* genes are located near the breakpoint region of the *MLL* fusion gene on chromosome 11q23 and are potential fusion partners with the *MLL* gene in ALL cells [26], [27]. In a recent study, the *MY03A* and *DBC1* genes were included as methylated markers a panel of 10-genes for detection of bladder cancer in urine samples [28], which is interesting in light of mounting evidence for generalized differentially methylated regions across different cancer types [29]. Besides *DBC1*, which is a suspected tumor suppressor gene [30], the precise functions on the molecular level of the other genes highlighted in our study have not yet been defined in ALL.

**Table 3:** Functions of genes with differential methylation between acute lymphoblastic leukemia cells and normal bone marrow cells.

Gene symbol^a^	Gene name	Cellular function^b^
*ACCS*	1-aminocyclopropane-1-carboxylate synthase homolog (Arabidopsis)(non-functional)	biosynthetic process
*ACY3*	aspartoacylase (aminocyclase) 3	metabolic process
*AMICA1*	adhesion molecule, interacts with CXADR antigen 1	cell-to-cell signaling and interaction*, adhesion, leukocyte transmigration
*CD300LF*	CD300 molecule-like family member f	hematological system development and function
*COL6A2*	collagen, type VI, alpha 2	apoptosis*, adhesion, cell cycle
*CPNE7*	copine VII	lipid metabolic process, transport
*CR1*	complement component (3b/4b) receptor 1 (Knops blood group)	cell death*, cell-to-cell signaling and interaction*, adhesion, hematological system development and function
*DBC1*	deleted in bladder cancer 1	cell death*, cell cycle
*EYA4*	eyes absent homolog 4 (Drosophila)	cell death*
*FAM83A*	family with sequence similarity 83, member A	NA
*FXYD2*	FXYD domain containing ion transport regulator 2	growth and proliferation
*LGALS8*	lectin, galactoside-binding, soluble, 8	cell death*, cell-to-cell signaling and interaction*, adhesion
*MYBPC2*	myosin binding protein C, fast type	adhesion
*MYO3A*	myosin IIIA	NA
*PI16*	peptidase inhibitor 16	NA
*RUNDC3B*	RUN domain containing 3B	NA
*RYR3*	ryanodine receptor 3	cell-to-cell signaling and interaction*
*SEC14L4*	SEC14-like 4 (S. cerevisiae)	transport
*THSD7A*	thrombospondin, type I, domain containing 7A	NA
*TMEM2*	transmembrane protein 2	NA
*UQCRFS1*	ubiquinol-cytochrome c reductase, Rieske iron-sulfur polypeptide 1	cell death*, cell cycle
*WDR35*	WD repeat domain 35	cell death*, cell cycle
*ZNF462*	zinc finger protein 462	NA
*ZNF502*	zinc finger protein 502	NA

a Gene symbol according to the HUGO Gene Nomenclature Committee (http://www.genenames.org/).

b According to Ingenuity Pathway Analysis (IPA);

* indicates enriched cellular function P<0.05; NA indicates undefined cellular function.

## Discussion

In our study of DNA methylation patterns in regulatory regions of 413 genes known to display ASE in ALL cells [4], we identified 24 genes with recurrent differential CpG site methylation that distinguished unequivocally between bone marrow cells from ALL patients and non-leukemic bone marrow cells. To control for possible inter-individual variation in DNA methylation patterns, we compared the ALL cells from each individual patient with “normal” mononuclear cells isolated from bone marrow of the patients during follow-up of the treatment when the patients were in remission. We also included bone marrow and blood cells from non-leukemic control individuals in the comparison. It should be noted that the diagnostic ALL samples contained ≥90% lymphoblasts, while the samples at remission and the samples from the non-leukemic control individuals consist of mononuclear cells from all normal hematopoietic cell lineages, i.e. lymphoid, myeloid and erythroid progenitor cells at varying stages of differentiation. The methylation patterns of the bone marrow cells from the patients at remission and the non-leukemic controls were indistinguishable from one another, and clearly distinct from the methylation patterns in the ALL cells at diagnosis. We recognize that the exact proportion between the mononuclear cell types may have varied between the individual remission or control samples. Yet, the biological roles of the differentially methylated genes justify that they could be further explored as diagnostic markers for ALL.

Our hypothesis when selecting the 413 genes for methylation analysis based on ASE analysis was that hypermethylation of CpG sites in gene promoter regions may cause ASE by silencing the expression of one of the alleles of expressed genes, and that hypomethylation of one allele could allow expression of only one of the alleles of a gene. ASE can be one-directional, so that all individuals over-express the same allele, or bi-directional, so that either of the two alleles may be over-expressed in different individuals. The majority of the CpG sites in **Table 2** displayed methylation differences with absolute Δβ-values near 0.5, which could reflect complete methylation or lack of methylation of a CpG site on one of the alleles of a gene in the individual ALL cells, as opposed to complete or no methylation of the corresponding CpG site in the normal cells. Of the 22 genes identified in the present study for which ASE data was available, 73% (16/22) displayed bi-directional ASE in ALL cells [4], indicating that stochastic methylation or de-methylation of either allele could cause ASE. Our study confirmed the ALL-specific hypermethylation of three genes (*DBC1*, *RUNDC3B*, and *COL62A*) [23], [24], [25]. Eight of the differentially methylated CpG sites identified here have been included subtype-specific classifiers for ALL (Table S2) [11]. Although the methylation levels for these sites differs between ALL subtypes, the methylation levels of 27 out of 28 of the sites in the BCP and T-ALL samples deviated from the bone marrow samples at remission in the same direction, indicating that most of these CpG sites reflect “global” ALL-specific changes independent of subtype. The CpG site that was differentially methylated between ALL immuno-phenotypes is located in the *MYBPC2* gene and is previously known for distinguishing between BCP and T-ALL [11]. Furthermore, eight of the genes (*COL6A2*, *EYA4*, *MYO3A*, *RUNDC3B*, *RYR3*, *SEC14L4*, *ZNF462*, and *ZNF502*) were highlighted in our previous study as potential markers for clinical outcome in two subtypes of ALL [11]. Thus, it appears that the aberrant methylation in these genes was acquired in the ALL cells, which renders them potentially interesting targets for studying the molecular events that lead to ALL. According to pathway analysis, the genes identified here are enriched for important cellular functions like cell-to-cell signaling and interaction or apoptosis (*P*<0.05). The majority of the genes that we identified in our study are hypermethylated in the ALL cells compared to controls, and for 9 out of the 20 genes for which published mRNA expression data from ALL cells was available [16], [17], the methylation levels determined in our study show evidence for an inverse relationship with gene expression.

We conclude that our candidate gene approach based on an initial genome-wide survey of ASE in ALL cells was a viable approach to zoom in on genes with methylation signatures that are characteristic of ALL cells and that have plausible functions for the development of ALL. Whether the aberrant methylation patterns in ALL cells were acquired stochastically or is an epigenetic mark characteristic of the leukemia initiating cell [31] will be a key question to address using new tools for genome-wide methylation analysis in future studies.

## Supporting Information

Figure S1 **Boxplots showing validation of the GoldenGate Assay by Sanger sequencing.** Bisulfite-converted DNA from eight ALL samples was PCR amplified and sequenced at five randomly chosen CpG sites in five genes (*ZNF502* chr3:44,729,363, *TNIK* chr3:172,661,831, *LOXHD1* chr18:42,435,264, *NOTCH3* chr19:15,172,990, and *NKAIN4* chr20:61,357,043). The methylation status of the C nucleotide in the CpG site as detected by Sanger sequencing (horizontal axis) is plotted against the Beta-values measured by the GoldenGate assay (vertical axis). The data is from Milani *et al.* [11].
(PDF)

Table S1 Data across 1,320 CpG sites for all samples included in the study.
(XLSX)

Table S2 Reproducibility of the DNA methylation analysis and the methylation levels of the 28 CpG sites with differential methylation according to ALL immuno-phenotype.
(XLSX)
