# Supplementary figures and images for: DNA Methylation Analysis of Bone Marrow Cells at Diagnosis of Acute Lymphoblastic Leukemia and at Remission
Source: PLoS One. 2012 Apr 6;7(4):e34513. doi: 10.1371/journal.pone.0034513 (PMC3321015; doi:10.1371/journal.pone.0034513)

Supporting Information

Figure S1.

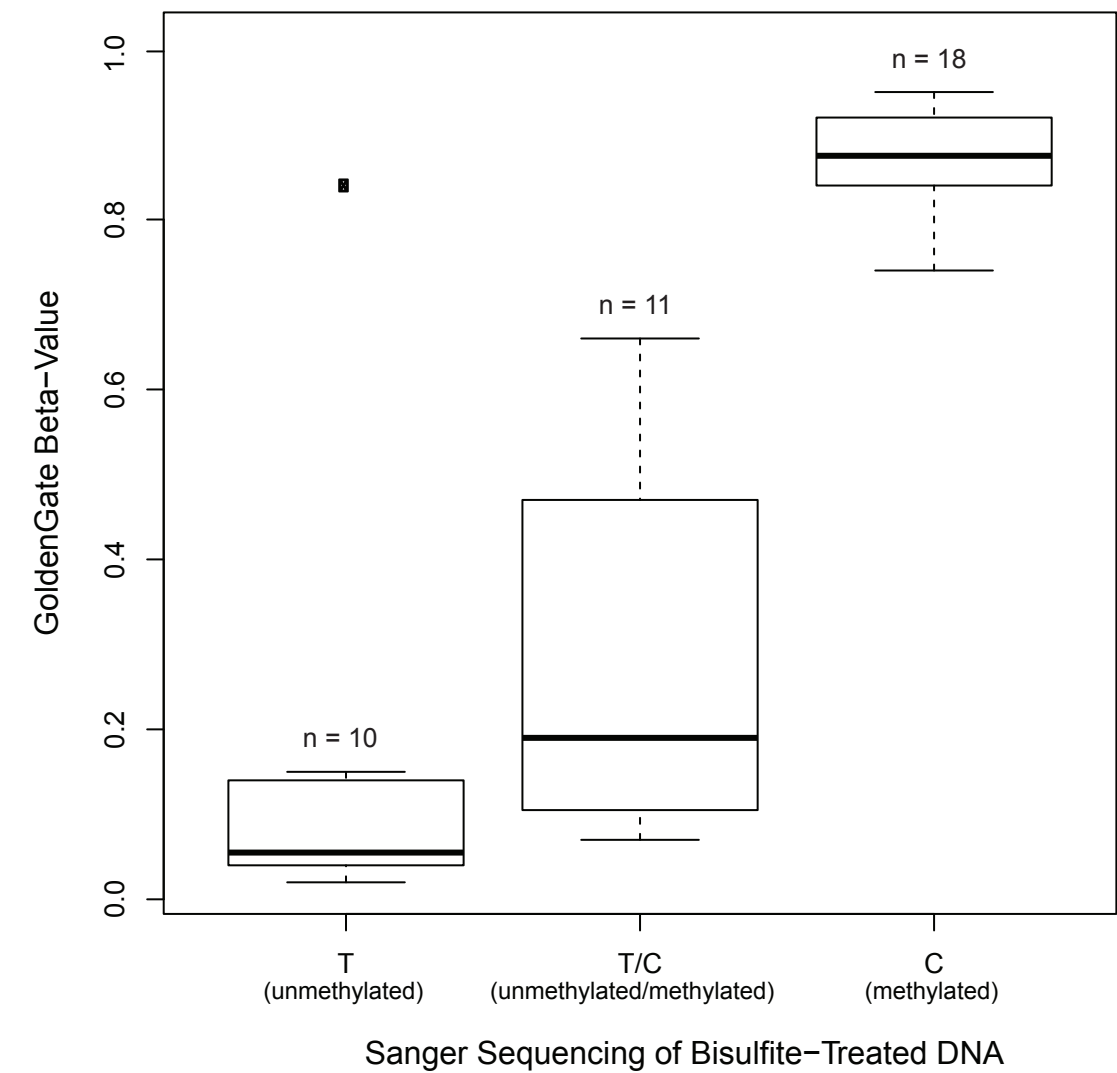

Supplement: Figure S1 — Boxplots showing validation of the GoldenGate Assay by Sanger sequencing. Bisulfite-converted DNA from eight ALL samples was PCR amplified and sequenced at five randomly chosen CpG sites in five genes (ZNF502 chr3:44,729,363, TNIK chr3:172,661,831, LOXHD1 chr18:42,435,264, NOTCH3 chr19:15,172,990, and NKAIN4 chr20:61,357,043). The methylation status of the C nucleotide in the CpG site as detected by Sanger sequencing (horizontal axis) is plotted against the Beta-values measured by the GoldenGate assay (vertical axis). The data is from Milani et al. [11]. (PDF) [file pone.0034513.s001.pdf]
